# The Unseeing State: How Ideals of Modernity Have Undermined Innovation in Africa’s Urban Water Systems

**DOI:** 10.1007/s00048-017-0160-0

**Published:** 2017-03-01

**Authors:** David Nilsson

**Affiliations:** Division of History of Science, Technology and Environment, KTH Royal Institute of Technology, Teknikringen 74 D, 100 44 Stockholm, Sweden

**Keywords:** Technological change, Social Construction of Technology (SCOT), Innovation, Closure, Africa, Urban infrastructure, Water and sanitation infrastructure, Technischer Wandel, Social Construction of Technology (SCOT), Innovation, Schließung, Afrika, Städtische Infrastruktur, Wasserversorgung, Abwasserentsorgung

## Abstract

In contrast to the European historical experience, Africa’s urban infrastructural systems are characterised by stagnation long before demand has been saturated. Water infrastructures have been stabilised as systems predominantly providing services for elites, with millions of poor people lacking basic services in the cities. What is puzzling is that so little emphasis has been placed on innovation and the adaptation of the colonial technological paradigm to better suit the local and current socio-economic contexts. Based on historical case studies of Kampala and Nairobi, this paper argues that the lack of innovation in African urban water infrastructure can be understood using Pinch and Bijker’s concept of technological closure, and by looking at water technology from its embedded values and ideology. Large-scale water technology became part of African leaders’ strategies to build prosperous nations and cities after decolonisation and the ideological purpose of infrastructure may have been much more important than previously understood. Water technology had reached a state of closure in Europe and then came to represent modernisation and progress in the colonial context. It has continued to serve such a similar symbolic purpose after independence, with old norms essentially being preserved. Recent sector reforms have defined problems predominantly as of economic and institutional nature while state actors have become ‘unseeing’ vis-á-vis controversies within the technological systems themselves. In order to induce socio-technical innovation towards equality in urban infrastructure services, it will be necessary to understand the broader incentive structure that governs the relevant social groups, such as governments, donors, water suppliers and the consumers, as well as power-structures and political accountability.

## Zusammenfassung

Verglichen mit europäischen Strukturen der Daseinsvorsorge stagniert die konstruktiv-technische Entfaltung der Infrastruktursysteme in afrikanischen Städten, sodass sie dem Leistungsbedarf nur unzureichend gerecht werden. Während der Anschluss an urbane Wasserversorgungsstrukturen hier privilegierten Kreisen vorbehalten bleibt, fehlt es ärmeren Stadtteilen zu oft an Zugängen zu grundlegenden Versorgungsleistungen. Auf Grundlage zweier historischer Fallstudien (Kampala und Nairobi) argumentiert der Beitrag, dass sich die unzureichenden Anpassungsleistungen urbaner Wasserversorgungsinfrastrukturen an ihr sozio-ökonomisches Umfeld mit dem Konzept technological closure durchdringen lassen. Um die vorherrschenden Strukturen zu verstehen, bedarf es zudem einer näheren Untersuchung der ihnen eingeschriebenen Werte und Ideologien. Als großtechnische Infrastruktursysteme im Zuge der Dekolonisierung als Sinnbilder des technischen Fortschritts in Afrika Verbreitung fanden, hatten diese in Europa bereits den Status einer diskursiven Schließung („closure“) erreicht. Auch nach der Unabhängigkeit erfüllten sie weiterhin vor allem symbolische Funktion, während staatliche Akteure die Kontroversen um die Versorgungsprobleme weitgehend ignorierten. Rücken wir die maßgeblichen sozialen Akteure – Regierungen, Geldgeber, Wasserversorger und Nutzer – wie auch die Macht- und Anreizstrukturen in den Mittelpunkt der Analyse, wird es möglich, das Verständnis für diese (post)kolonialen Eigendynamiken zu vertiefen.

## The Puzzle of Technological Change in African Cities

Is poor infrastructure really the problem in African cities? Or is it our inability to see and think about technology in new ways that keeps millions of people from having access to safe water and decent sanitation services? Water infrastructures—both their technical as well as their social components—are made, operated, and used by people. Being man-made systems, infrastructures may be moulded to fit varied social purposes with different setups fitting different contexts. But what if the people who finance, design and construct infrastructures can only envision specific setups of these systems? What if the social purpose of water infrastructure is not merely to provide water, but to exert power, legitimise a social order, or simply give an impression of progress? What is then the prospect for change?

This paper discusses changing dynamics of socio-technical systems for water in African cities south of the Sahara in a post-colonial context using historical case studies from Kampala in Uganda and Nairobi in Kenya. More specifically, the aim is to illustrate how change and adaptation from the 1960s and onwards have been affected by coupling the Northern-style large-scale water systems closely to the ideals of modernity and progress. The pursuit of modern ideals led colonial administrators, engineers and city-builders to import European water technology and institutions, a process that also prompted state actors to apply a simplified view on the African social and physical environments. The persistence of these ideals over time and the continuity of state simplifications, I argue, have had a negative effect on search and innovation activities in the socio-technical systems, to the extent that key system-builders (typically state representatives at various levels) are largely unable to see critical problems that need to be solved. Hence, not only is it relevant to indicate how government actors in East Africa adopted the certain kind of “seeing” which was so characteristic of the high modern state (Scott 1998). It can also be claimed that state actors in Africa have become ‘unseeing’ with respect to innovation needs in water infrastructure, as they view their worlds through the lenses of Western modernity, to which ready-made solutions can be imported and applied.

To understand change and continuity in these systems in Africa, I believe that it will be necessary to lay bare and engage with the ideological content of urban water technology. The United Nations (2013) have called for a “transformative change” to usher in global sustainable development, and if we are to take this seriously, we need to widen how we think and talk about technology in development settings. We must also consider how attitudes, ideals and political aspirations create a landscape that affects the possibilities of change. Being able to deliver progress and modernity, or rather, seemingly deliver progress and modernity has been a real concern for political leaders across Africa since independence. The aim of my paper is to explore how this impacted the shaping of urban infrastructure. Understanding this process, I believe, will not only be of interest for historians or students of technological change. Hopefully it could also prove to be important for development actors and policy-makers en route towards implementing the human right to water and sanitation.

Half a century has passed since a majority of states in sub-Saharan Africa gained formal independence from their European colonisers. Important strides have been made over the decades and the quality of life has improved for millions of people. Several African countries have met the water targets of the Millennium Development Goals of halving the proportion of people without safe water from 1990 levels, including poor countries like Ethiopia, Malawi, Mali and Uganda (UNICEF & WHO 2015). But the picture is lopsided and the advancements over the past decade have in many cases been made from a very low starting point. In regions like East Africa, access to water services had even deteriorated in the thirty years following independence (Thompson et al. 2001). The development on the African continent needs to be seen within the context of the huge social transformation currently witnessed, what Sue Parnell and Edgar Pieterse (2014) have termed Africa’s “urban revolution”. All over Africa, pressure on cities from rural-urban migration and population growth has been enormous. Africa’s urban population grew from 54 million in 1960 to 395 million in 2010; a staggering 630 per cent increase over fifty years (UN 2014). During this large-scale social change process, formal systems for service provision have lagged behind. In Kenya, the official coverage figures for urban water supply dropped from a near 100 per cent in 1963 to 53 per cent in 2009.^1^ Similar pictures of an urban infrastructure development lag have been observed all over the continent. A regional analysis carried out by the World Bank in 2011 concluded that “[i]nvestments in urban water supply have not kept up with urbanization and population growth.” (van Ginneken et al. 2011: viii). But the problem of deteriorating water services in African cities cannot be reduced to a question of lacking funds. While it is true that in African countries public finance is generally limited, the same regional study identified no relation between levels of public spending and the levels of access to water supply and sanitation in the different countries.

For the past 25 years, aid donors have pressed water sector reforms in Africa south of Sahara to improve performance and to attract investment, often with a privatisation agenda (Bayliss 2003). Not surprisingly, heated debates have raged among scholars, policy-makers and corporate leaders regarding the economic versus social nature of water: should water be regarded as a private commodity or as a social good?^2^ Typically these reforms have served to improve financial viability of the existing large-scale systems, but not to change their configurations. From an equity or poverty-reduction point of view the donor-led sector reforms in the 1990s and 2000s were thus not very helpful. The urban poor generally did not have access to the large-scale piped water systems and relied instead on alternative, informal and small-scale service provision (Collignon & Vezina 2000; Dagdeviren & Robertson 2009). While privatisation attempts sparked violent debates, Jessica Budds and Gordon McGranahan (2003) have shown that these debates largely missed a crucial point; most of the water accessible for the urban poor was already in private hands. This “privatisation by default” has not taken place as part of any reform agenda, but rather as a response to the failure of the public service provision (Kjellén 2006). Although the United Nations declared access to water and sanitation a human right in 2010, putting this into practice still remains an issue under formation (Albequerque & Roaf 2012).^3^ Several countries in Africa have introduced a rights-based approach to water as part of their sector reforms, but huge difficulties persist in the implementation of these rights (Drakenberg & Nilsson 2015; Plessis 2010).

Where is technological change in the reform talk? There has been no lack of awareness regarding the need to find practical and affordable solutions in developing countries. There was considerable buzz regarding ‘appropriate technology’ and ‘social carriers’ in the 1970s, followed by the outcry over ‘white elephants’ in the 1980s.^4^ Despite all the awareness-raising, surprisingly little attention has been paid to the mechanisms of socio-technical change in Africa by scholars and policy-makers. The past decade has seen a growing number of studies on history and socio-technical change in Africa’s urban water systems. A conference in South Africa in 2004 gauged the burgeoning activity on African water history all over the continent (Tempelhoff 2005). Important contributions have since been made on Nigeria (Gandy 2006) and Ghana (Bohman 2010), on Kenya and Uganda (Nilsson 2006a; 2011; 2013; Nilsson & Nyangeri 2008) and Zimbabwe (Musemwa 2008; 2010), to mention but a few. In addition, a range of studies on water resources and dams exists (Showers 2011). This body of literature is still very small compared to European or North-American contexts. Moreover, a lot of the work has been done by non-African scholars, which raises questions about the applicability of Northern theory on African cities (Furlong 2014). Theories based on the cultural and historical experience of countries of the global North must be critically adapted and carefully situated in African contexts (Lawhon et al. 2014). Understanding context is therefore essential. In 2011 I argued that Large Technical Systems (LTS) theory can explain the historical trajectory of urban water systems in East Africa—but only if the informal character of African cities is understood as a ‘reverse salient’; a barrier that stops the larger system from growing. Similar arguments can be made about urban transport in Africa. Transport monopolies established in the colonial period have been more or less replaced by small-scale informal operators with bikes, motorbikes and minibuses, due to the inability of the large-scale public transport utilities to adapt to informal and low-income realities of African cities (Behrens et al. 2016).

What is obvious today, at any rate, is that Africa has not witnessed the continuous expansion of infrastructure observed in European cities a century ago. Infrastructure systems in Africa do not follow neatly in the footsteps of the North. And why should they? Historians and urban analysts are perhaps too quick to compare the cities of Africa with those of richer countries, and by doing so erecting a normative position and an intuitive frame of analysis. Fourchard points out: “Considering Africa’s cities as dysfunctional, chaotic, failed, informal, or not globalized works to retain the Western city as the paradigmatic model against which all others are to be assessed.” (Fourchard 2011: 247).

Technological change in African cities largely remains a puzzle. We need to understand the conservative forces that seem to hold back development of sustainable services, preventing innovation and adaption. We also need to identify the forces that stimulate change and innovation. This entire special issue makes important contributions to our knowledge of socio-technical change in the South. Within the themes outlined in this issue, my paper adds a distinct perspective on imperial diffusion of technology within the British Empire: that of modernisation. The association of urban technology with modernisation turns our attention from technology as a tool of conquest towards technology as a tool of social order and progress, and as a vehicle for ideology (see Hasenöhrl and van der Straeten, this issue). When public infrastructure comes packed with ideology, citizens can hardly avoid the effects of these ideologies. Technological systems of water thus transcend the public realm far into the private sphere of households and individuals. The second contribution that this paper makes to the debates contained in this special issue is to what extent Northern theory—especially LTS and Social Construction of Technology—can be useful for analysing and explaining infrastructure development in Africa. In this respect, my main focus is on how change dynamics are affected by ideology, state simplifications and power structures.

In section two, I will outline how modern water technology in Europe fused with ideals of progress and modernity over centuries. These socio-technical configurations then diffused through imperial structures in the late nineteenth and early twentieth century, which I look at in section three. Thereafter I discuss in more detail the case of the Kenyan capital Nairobi, and how infrastructure and ideals of modernity were construed in the transition from a British colony to an independent state. Sections three and four are to a large extent based on archival material from the Kenya National Archives in Nairobi and the archives of the Colonial Office at the National Archives in London. In the fifth section I return to the discussion on change dynamics where I combine strands of thoughts from LTS with Pinch and Bijker’s (1987) concept of technological closure, which describes the state in a technological design process when social groups regard the problem as being solved and thus “close” it for alternative designs. Finally, I will argue in my closing remarks that innovation and change have been held back by African social elites and by donors, who have favoured the preservation of ideals coded into existing water technology centuries ago—those of modernity and progress.

## Packing Modernity into Pipes

Progress is not a neutral term; it moves towards specific ends, and these ends are defined by the possibilities of ameliorating the human condition. (Marcuse 1962: 18).

Herbert Marcuse (1898–1979) wrote *The One-Dimensional Man* in 1962 but his text still bespeaks the vast power of technology as a mould of human thinking and acting. What we believe is right and possible is to a large degree coded into and fed back to us from technological systems and artefacts surrounding us, creating a one-dimensional and rational universe of what progress means. Technological rationality, he argued, despite its claims for objectivity and universality, remains essentially within the realm of politics. Since then, a vast field of inquiry has emerged in technology studies, which has seen heated debates on the political nature of technology.^5^ Not only is technology a representation of values, but also extends and offers ways of pursuing political ends and promoting ideologies (Hecht 1998; Edwards & Hecht 2010). Similarly, ideology can be the starting point for developing a technological practice. In *Seeing like a State*, James Scott argues that the modern European city—with Paris as the monumental showpiece—evolves out of the state’s need to improve performance of urban functions and to exert stronger political and military control (Scott 1998). In the course of transforming the nineteenth century city, state actors and urban planners reduced complexity by simplifying, as well as standardizing, social and physical structures in order to make them legible and controllable. These state-led processes of ordering nature and social life were often underpinned by ideological aspirations that Scott calls “high modernism”. Its flag bearers were typically “the avant-garde among engineers, planners, technocrats, high-level administrators, architects, scientists and visionaries” who all “envisioned a sweeping, rational engineering of all aspects of social life in order to improve the human condition.” (1998: 88). Naturally, the modernist manifesto permeated also the water infrastructure of cities.

The large-scale water and sanitation projects during European industrialisation and urbanisation attracted the attention of the public, epitomizing the new, modern, sanitary city (Melosi 2000). Against the backdrop of the social and sanitary movements championed by reformers like Edwin Chadwick and Joseph Chamberlain, the construction of the Thames embankment in the 1860s and 1870s was the talk of the London elite (Porter 1998). It was precisely this ability to mobilise influential and powerful groups in society around the cause of water, sanitation, hygiene and modernity that was so crucial for the rapid development of water infrastructure in European cities (Szreter & Woolcock 2004). Geels (2006) also showed that in addition to the centrally placed administrators and planner, rather externally placed actor groups such as medical doctors and hygienist reformers were quite influential in introducing water and sanitation infrastructure. Water and sanitation became associated with ideas of purity, salubrity, and morality (Roberts 2006). Public health and disease had in the early 1800s primarily been described as a moral problem and thus a problem for the individual (Reid 1991). But with the growing understanding of contagious processes in the mid 1800s, first the *miasma* theory, and later the discovery of bacteria, public health was increasingly regarded as a social problem (Melosi 2000). It is clear that the forces contributing to the establishment of modern water and sanitation in European towns were many. The fear of cholera outbreaks; water needed for fire-fighting and industrial use; the drive for societal modernisation and increased social equity were all among the important factors (Anderson 1988; Goubert 1989; Hallström 2002; Hamlin 2009).

This condensed overview of the formation of urban water as a symbol of progress and modernity in Western society might not seem to have much bearing on Africa. However, this is an immensely important prehistory to the age of imperial technological transfer. We need to understand the ideological content of technology, especially since urban water is of a highly political nature (Swyngedouw 2009). If we historians, policy-makers, and system-builders overlook this prehistory of how technology became packed with ideology, it reduces our understanding of what these systems are and what they mean. We will not see what needs to change in our thinking. In the terms of Marcuse: we are devised to think in one dimension; within the bounds demarcated by the existing and self-reproducing technological paradigm.

## Signed, Sealed, Delivered: Exporting Modernity

The surplus capital of Western societies combined with a need for precious natural resources and new markets drove the frontier of the industrial world outwards from the 1870s (Barbier 2011). As Europe expanded its imperial influence over the southern hemisphere so did also the reach of its technologies. Northern technology was in itself a prerequisite for conquest of distant territories (Headrick 1981). Technology acquired symbolic value within the imperial order, showcasing the colonisers’ superiority and creating legitimacy of the occupation as it contributed progress and civilisation (Adas 1989). Technological transfer to the colonies became a way of demonstrating power, while at the same time creating small enclaves of European ideals, technologies and ways of life for the ruling (white) élites in the cities (Headrick 1988).

The export of modern ideals in the form of infrastructure took place in virtually all the major European powers’ colonial machineries. In the Dutch East Indies the colonial administration built a first water supply for Batavia (now Jakarta) in 1873, to supply its 8,000 white residents with clean water while the local Indonesian population was denied its access. The ‘natives’ using unclean river water were regarded as ‘backwards’ while the European elites were construed as ‘civilised’ and modern (Kooy & Bakker 2008). Similarly, when the British colonial administration introduced sanitary measures in Bombay in the mid-1800s, British norms, practices and cultural preferences were the point of reference for technological solutions. Improving sanitation in colonial Bombay was not just a matter of transferring technology, but to spread a Victorian ideal of purity (MacFarlane 2008). In French colonial cities, such as Dakar, service provision was often racially segregated based on the concept of the cordon sanitaire (Njoh 2007). In the French settlement Saint-Louis du Senegal in West Africa, the colonial government installed a piped water system in 1886 covering mainly the European part of the town. During a cholera outbreak in 1893, about thousand Africans died—but only four Europeans. The French medical officer in the colonial administration concluded that the reason for Africans being hit much harder were their “unsanitary habits”—and not their limited access to safe water (Ngalamulume 2005). Cleanliness, water supply and sanitation thus became part of a narrative constructed around race, modernity and development in the late nineteenth century, which extended throughout the European imperial project.

A comprehensive analysis of British colonies in Africa is clearly beyond the scope of this paper, but I will offer an illustrative example from one of Britain’s colonial prizes. Uganda was known at the time of its colonisation as ‘the pearl of Africa’. Kampala, today the capital of Uganda, was a divided town during the colonial period. One part—the Kampala township—was administered by the British Protectorate Government and the other part—the Kibuga—by the King of Buganda (Zwanenberg & King 1975). In the first decades of the nineteenth century, water was provided mainly through means of rainwater, which was harvested and stored in tanks, complemented with surface spring water in the dry season. The sanitation services consisted of a “single bucket” latrine system, where faeces were collected from the households at night and buried in trenches outside of the town.^6^ In 1913, Professor William John Simpson (1855–1931), a sanitary expert sent out from the Colonial Office, recommended the introduction of a piped water supply for Kampala as the local sources were not considered safe.^7^ The colonial government in Uganda, which was located in Entebbe, carried out a technical investigation in 1924, proposing a piped scheme from Lake Victoria 12 kilometer away, designed to supply mainly the European and Indian populations in Kampala township, and possibly areas in the Kibuga. Already at this conceptual stage, the water engineer of the Public Works Department in Entebbe, W.G. Morris, envisioned a large-scale system that should be able to cater for “an ample consumption per head to the non-African population in Kampala” to allow for the subsequent installation of a piped sewerage system.^8^ The per capita design demand was therefore set to 180 litres per day for the European and Asian population, while the water consumption of the few Africans living inside Kampala township boundaries was estimated half of that.^9^ The Colonial Office back in London, however, was not particularly enthusiastic. The anticipated high cost of piped water supply was particularly troublesome, and the Colonial Office stated that it would be “worthy of consideration whether the collection and storage of rain-water could not be utilised to a greater extent”.^10^ The Entebbe administration discarded this alternative on water quality and public health arguments, supported by statistics of cholera outbreaks provided by the medical officer.^11^ Another alternative to the Lake Victoria supply was investigated: a number of boreholes were drilled near Kampala in 1926. The engineers in Entebbe argued, however, that the new water source should ideally be able to provide 22,500 cubic meter per day, or at least 13,500 cubic meter per day, a criterion that the boreholes could not satisfy.^12^ This was a strong rational argument, but it should be noted that the amount of 13,500 cubic meter exceeded about ten times the actual water demand, which had been calculated at 1,370 cubic meter per day.^13^ In the face of the high aspirations and ambitions expressed by health experts, engineers and the colonial administrators in Uganda, the large-scale solution of piping water from Lake Victoria appeared as the logical, or maybe sole solution. After years of negotiations, the colonial administrators and experts in Entebbe eventually succeeded to convince their counterparts in London. In 1928 the Colonial Office approved the construction of a piped water supply for Kampala focussing on the European areas.^14^ Within a few years town-planning measures followed, and in 1936 works started on the main drainage and a piped sewer system in Kampala.^15^

The conception of the water and sewerage systems had been made by a handful of colonial decision-makers and engineers without much consultation with the people in Kampala and the Kibuga. The hopes expressed by Morris in 1924 that also the people in areas adjacent to the ‘white’ Kampala would become paying customers became already elusive in 1927.^16^ When the piped water supply opened in 1930 it was primarily designed to suit the needs of Europeans. While the large expenses for water and sewerage were officially justified on grounds of public health, these projects were clearly also driven by the colonialists’ quest for modernisation. Once the idea of a modern, European-style water supply and sewerage system had emerged as a real possibility among the experts and administrators in Entebbe, no other solution appeared plausible, and henceforth their attention focussed on convincing the Colonial Office in London. As Governor William Frederick Gowers (1875–1954) concluded in 1929: “[M]uch remains to be done if Kampala is to extend on the lines of a modern township”.^17^

The case of Kampala demonstrates the negotiated process of exporting modernity within the British Empire. It also illustrates the implications for post-colonial development. As the system was designed for the needs and preferences of Europeans, its services were not affordable for Africans and service provision became racially segregated. The ideals and norms transferred from Britain, through expertise and administrators, prompted a large-scale development (Nilsson 2006a). After the establishment of the large-scale, capital intensive systems in Kampala, the public administration of Kampala would carry a vastly increased recurrent cost for a long time, which created an in-built tendency to focus on the more lucrative customer segments. For decades, focus remained on serving the middle and upper-class customers in Kampala (Appelblad Fredby & Nilsson 2013).

The transfer of the modern ideal through city-builders in the colonies in Africa would hold a bad seed for development after independence, when the inherited colonial technological machinery got out of tune with finances, technical capacity and the average customers’ ability to pay. In his study of Lagos in Nigeria, Matthew Gandy points out that what remained after decolonisation was an ‘incomplete modernity’ (2006: 375) aspiring to the values, infrastructures and service levels of Europe, but which in reality was little more than a thought-figure. In the following, I will look in more detail at how this incomplete modernisation played out in city-building and water provision in the Kenyan capital Nairobi.

## Nairobi: A European City Close to the Equator

Nairobi as a largely European city situated close to the Equator is almost unique among the cities of the world. [...] Considering the advances made in sanitary science, [...] it would be deplorable if all possible advantage were not taken of modern science to render Nairobi at a comparatively insignificant expense, a model of a sanitary tropical city.^18^

Nairobi had been established as a railway depot halfway from the Indian Ocean to Lake Victoria in 1899 (Hill 1949). The city grew rapidly and between 1907 and 1913 a number of expert commissions were set up to solve mounting problems of city planning, public health, sanitation and water.^19^ The above quote clearly illustrates that colonial Nairobi was primarily seen as a European town and not an African one. Hence, the commissions typically sought practical ways for the realisation of European-style modernisation and progress, conferring onto the colonial capital an image of a model city, a beacon of civilisation. Over the decades up until independence in 1963, Nairobi grew from a small township of a few thousand, to a city of more than 300,000 (Nilsson 2011).

The water supply was expanded in several stages to meet the growing demand of the population. The Uganda Railways administration had built the first water supply to Nairobi around the turn of the century. Water was piped by gravity from the Kikuyu springs roughly 20 kilometer away and in 1907 Professor Bransby Williams, an expert sent out by the Colonial Office, estimated that the Kikuyu springs could yield up to 2,700 cubic meter per day.^20^ Throughout the 1920s, the growing population of Nairobi was repeatedly confronted with serious water shortages prompting the municipality to enforce rationing and strict water conservation measures.^21^ Hence the opening ceremony of a new water supply from the Ruiru river on November 16^th^ 1938 was surely a welcome event.^22^ The design by the British engineering firm *Howard Humphreys & Sons* comprised of a dam, modern filtration, and a gravity pipe through which water was led to Nairobi over a distance of 28 kilometer.^23^ Yet the relative abundance of water was not long-lived. Already in 1945 Francis Edgar Kanthack (1872–1961), a South African consultant and engineer, had been asked to assess the possibility of augmenting the water supply to Nairobi. The total supply at that time reached 9,000 cubic meter a day, which was considered “quite inadequate”.^24^ A drought in 1946 made things even worse and the municipality quickly built a dam along the Ngong River near Nairobi and expanded the Ruiru scheme.^25^ Based on Kanthack’s proposal, a new dam was built at Sasumua on Chania River, which started supplying water to the city in 1956. The new supply promised to add another 18,000 cubic meter per day, thus alleviating Nairobi’s water shortage for yet another number of years. Again water was piped by gravity from the Kenyan highlands over a distance of 64 kilometer.^26^ In terms of water supply Nairobi’s colonial period was on the whole a story of constant search for more water to supply the ever-thirstier city, rather than managing its demand.

During this expansion phase, British technology and design norms were used as far as the financial and technical capacities of the colonial administration allowed (Nilsson 2011). The design demand norm for water followed British norms so that Europeans living in Nairobi would be provided with more or less the same service level they were accustomed to at home, with design demands well over 200 litres per person and day. This did not, however, apply to the provision of the Asians and Africans living in Nairobi, for whom a considerably lower design demand was used (see Table 1). The promise of modernisation and progress of this ‘model city’ was not applied in equal measure to everyone. The racially biased water supply remained formally in service until Kenya’s independence in 1963. But as I will show, segregationist practices embedded in the city’s technological backbone would not so easily be undone.

**Table 1:** Design demand figures in colonial Kenya in 1934^27^

	Design Demand (litres per capita per day)
Europeans	225
Indians	135
“Substituted Natives”	90
“Natives”	68

With independence came the strong wish to quickly develop the young African nation. The political manifesto of the independent government—“African Socialism and its application to planning Kenya”—is replete with expressions of African nationalism and modernisation. Through modernisation and rapid development the young nation should be rebuilt and reborn:

Under colonialism the people of Kenya had no voice in government: the nation’s national resources were organized and developed mainly for the benefit of non-Africans […] The best of Kenya’s social heritage and colonial economic legacy must be reorganized and mobilized for a concerted, carefully planned attack on poverty, disease and the lack of education in order to achieve social justice, human dignity and economic welfare for all.^28^

Technology and infrastructure were crucial development factors, and the Kenyan government concluded that:

The ability of Africa to borrow advanced technological knowledge, modern methods of industrial organization and economic techniques of control and guidance from more advanced countries provides the opportunity to leap over many of the hurdles that have restrained development in these modern societies in the past. 29

As part of this larger process of African modernisation and development, the Kenyan government launched massive investments into water infrastructure (Nilsson & Nyangeri 2008). The government’s clearly stated objective was to provide every Kenyan with clean water by the year 2000.^30^

The development of water infrastructure—especially distribution systems—in the cities cannot be analysed isolated from city-building and housing development. At the time of Independence, the government faced a backlog of housing, infrastructure and services in the African areas, and already in the 1950s informal settlements had started to come up (East Africa Royal Commission 1955; Stren 1972). Through a National Housing programme commissioned by the Ministry of Housing, thousands of new housing units were planned in the late 1960s. However, the development of the new housing areas took time, and (not surprisingly) became much more expensive than anticipated, rendering the housing programmes way behind schedule. Meanwhile, informal settlement in the peri-urban areas accelerated, with makeshift houses and virtually no infrastructure (Mitullah 1999; Nilsson & Nyangeri 2008). For the new independent government it was politically very difficult to lower the infrastructural standards as it was at the same time constructing its image around African nationalism, progress and development (Stren 1972; Werlin 1973). Central government actors became the new proponents of the modern African city like the colonial administrators before them. Below, I will give an example of how this process played out on the ground.

In January 1969 the Nairobi City Council started preparing a development project to raise the standards of living in Mathare Valley, an informal area on the outskirts of Nairobi, home to approximately 30,000 people. The council had ventured into re-development schemes in other so-called slum areas before upgrading water, sewerage, roads and housing. However, this just resulted in locally shifting the problem, given that higher living costs forced poor people to move to another region within the city. The Council had to realise that the problem lay in the installation of services that was unaffordable for “the man with no means”.^31^ A collaborative project took form to improve conditions in Mathare Valley between the City Council, the Ministry of Housing and its National Housing Corporation, and other wings of the government. The Council undertook various investigations on site, and also collaborated with locally based organisations, like the *National Christian Council *of Kenya.^32^ For the City Engineer of Nairobi the solution lay in installing piped water, sanitation, and security and to build very simple tenant houses also affordable for the poorest. The critical part was, however, finding an affordable housing unit. In 1969 the City Engineer proposed a design using a concrete floor and iron sheet roof supported by a timber structure, with sisal covered walls. This promised to be the “cheapest type of [housing] unit yet designed”.^33^

The Ministry of Housing, however, took a different position. For one thing, it was imperative for them to remove the eyesore of Mathare Valley, which was considered as “a health hazard and a threat to social and political stability.” Therefore Paul Ngei (1923–2004), the Minister for Housing, directed that “the slum must be cleared by starting immediately upon the creation of a new and well planned housing estate”.^34^ The acute sense of urgency on the part of the Ministry was fuelled by the fact that the country was headed for elections, and President Kenyatta himself had insisted that the project got underway.^35^ Addressing the slum dwellers at a gathering in Mathare Valley on June 5^th^ 1969 Ngei declared: “You are citizens like any other and entitled to a good living.”^36^ There was, simply put, political pressure to deliver on the promises of progress and development.

The Ministry of Housing regarded the low-cost solutions proposed by the Nairobi City Council as inadequate and dismissed the timber and sisal houses at a meeting on May 29^th^ 1969. The Ministry ordered that the redevelopment of Mathare Valley should aim for a higher standard providing housing and services for “tenants who are able to pay,” while the “destitutes and unemployed” should be “resettled in other schemes, such as agricultural schemes etc”.^37^ The City Engineer’s department quickly came up with a new solution, which in their eyes “offered real promise. It met the ministry’s investment criteria, was likely to be within a reasonable cost discipline and would provide hygiene and a roof in the first instance.”^38^ It consisted of a simple open structure using concrete and timber while the walls would be for the tenants to construct with simple materials. “As economic conditions improve, permanent materials would replace the mud and wattle.”^39^ A very similar low-cost proposal was put forward by Mr. L. Laurenti, an adviser to the Ministry of Finance.^40^ And again, the Ministry of Housing rejected the ideas of a simple, low-cost housing solution. A compromise was found in planning for a few experimental houses of low-cost type. But for the City Engineer, the Ministry’s unwillingness to find a solution for the poor in Mathare Valley was all too obvious:

[Officers of the City Engineering Department] feel bound to state, however, that Government requirements in accommodation and standards of construction are such that the project is moving away from the economic means of the people in the Mathare Valley. The Ministry has stated that it will only build houses for those who can afford them. It appears that this group has been drastically reduced by the Ministry’s own requirements.^41^

The inability of reconciling the two different perspectives of ministry and council, with the former repeatedly vetoing the designs proposed by the latter, effectively stifled innovation in Nairobi low-cost housing. As a consequence, by 1974 only 22 houses had been built through so-called self-help schemes for poor people of Mathare, whose population had by then soared to 60,000.^42^ The government was largely unwilling to finance the experimental houses, just as the low-cost houses were considered “undignified for a modern urban housing scheme”, as Herbert Werlin had noted already in 1973. The snail-pace of expanding affordable housing in Nairobi thus also held back expansion of water and sanitation services, since these two processes were closely interlinked: without housing development, no expansion of the formal water services.

**Fig. 1:**
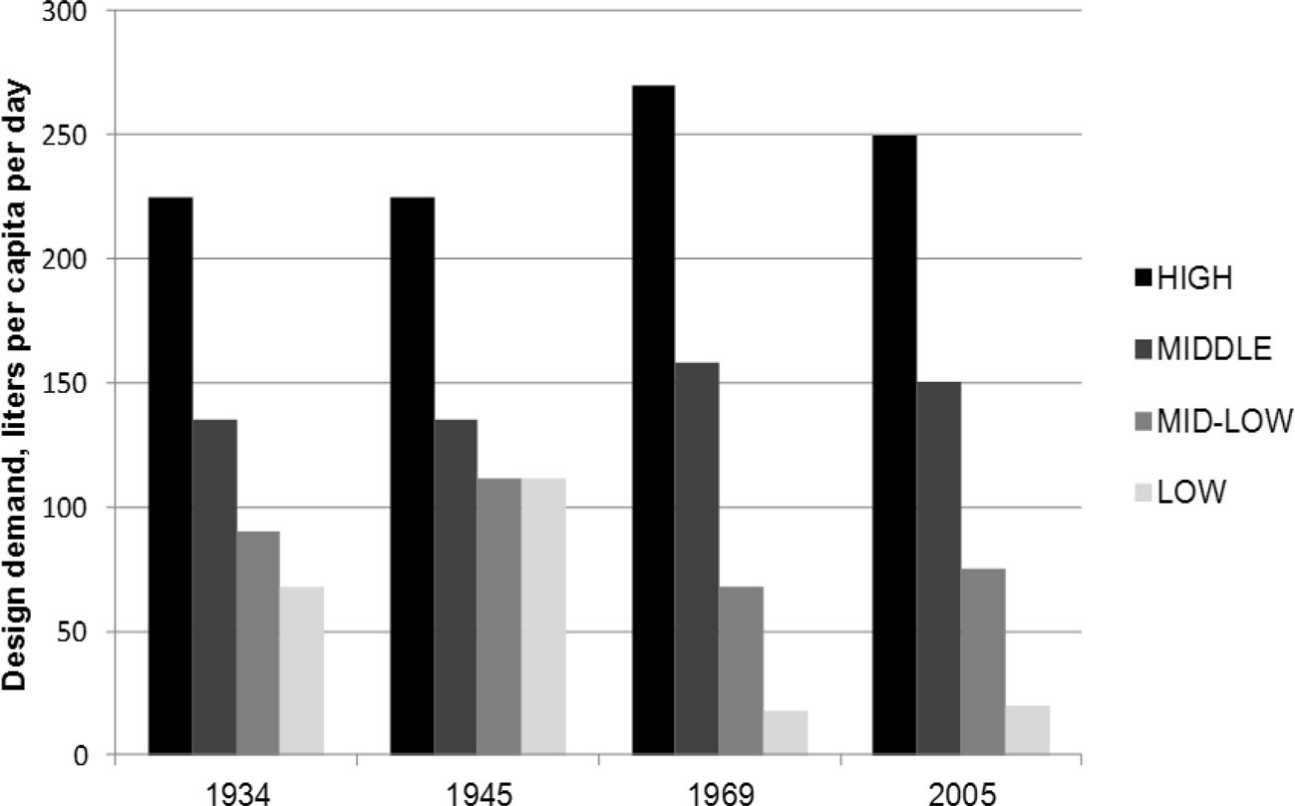
The per capita design norms for water in Nairobi, Kenya (adapted from Nilsson 2013: 172)

Another example of how innovation seems to have been held back by development ideals shaped earlier in the industrialised world is the “design demand” for water consumption in Nairobi (see Fig. 1). The design demand is a critical design parameter in any water development scheme as it basically sets the quantity standard of access to water. As previously discussed, the design demand was split along racial lines during the colonial period. After independence, the racial stratification was replaced by economic user categories like “high-income”, “low-income” and “squatters”.

This design parameter has been remarkably stable in Kenya since the 1930s. If any change can be discerned over time, it is that the lowest stratum (i.e. the poorest) has been assigned a lower design demand over time. The high-income group has been re-assured stable and high water consumption on a par with the European standards at well over 200 litres per person per day. But today Nairobi is a water-scarce city where the richest ten per cent of Nairobi’s population consume almost half of the water available in the city (Ledant et al. 2013). The rise of the total water demand is therefore to a large degree driven by this small and privileged consumer group. As described earlier in this section, there has been a continuous struggle to supply the city with more water from increasingly distant water sources since early colonial times. Currently, the water authority *Athi Water Service Board *is planning to bring water from the Tana catchment some 80 kilometer away, through a system of collectors and tunnels. The investment cost has been projected to over one billion USD, financed by loans from the World Bank and others (Nilsson 2013).^43^ From the above can thus be derived that this investment is driven by the decision-makers desire for a high per capita consumption, which in itself signals modernity. Another implication of the high consumption by the rich is that less water is available for the poor. Currently about one million people, that is, almost one third of the population, are not served by the public water company. The constitutionally protected right to water of every Kenyan remains to be implemented. But for those with money, Nairobi appears to be a largely European city situated close to the Equator; just as the colonialists had hoped for one hundred years ago.

More recent developments in East Africa confirm the trend that old colonial ideals hold back innovation. For example, an attempt to build ecological sanitation toilets in the early 2000s in informal settlements in Kampala, Uganda, failed due to outdated building regulations. The City Council, who implemented the project, insisted on using old building regulations from the colonial period. This meant that the cost of a single toilet unit amounted to around 2,5 MUSH (approx. 1,300 USD), which exceeded by far the means of a low-income household (Carlesen et al. 2008).^44^ It appears that in this case too, decision-makers were unwilling to change the technological standards established during colonialism, which have a severe and constraining influence on the provision of services.

## Closure and Innovation in Africa’s Urban Water

I now turn to the main question. How did the inbuilt ideology affect the change dynamics of urban water technology, and more precisely: could this have restricted the ability of state actors to ‘see’ what needs to change?

Being large (in terms of covering wide urban areas) socio-technical systems, one could expect modern urban water technologies to follow the LTS staged development as posited by Hughes (1983); with an establishment phase, followed by an expansion phase and as the system gains momentum, into stagnation in its mature phase. Yet as noted previously, when it comes to water development in African cities, some things do not add up nicely with theory. For instance, it can be questioned whether African urban water systems have expanded enough to make it susceptible to Hughes’ concept of momentum (Furlong 2014; Nilsson 2006b). LTS theory seems to fit certain parts of the historical trajectory of water networks in Africa, but not all (Nilsson 2011). What is puzzling is the lack of innovation in response to current challenges. The mismatch between local capacity, finance and cost levels of the established large-scale technologies has been apparent for decades, as well as the poor performance of the systems (Nilsson & Nyangeri 2008; Vaa 1993). Yet so little change has taken place in formal systems, and as illustrated above, key design norms and practices have been conserved since colonial times.

I propose that the lack of innovation and change in African urban water infrastructures can be explored using the concept of technological closure, a concept that gained wide recognition through Trevor Pinch’s and Wiebe Bijker’s classical paper (1987). If this concept feels dated to theoretically interested readers, it will be important to recall that it emerged from a debate in the 1980s about how actor groups influence innovation in technological systems, a debate which clearly aligns with the scope of this paper. According to Pinch and Bijker, closure means the end to a controversy (a design problem) in technological development. By trying out new solutions either through market-based competition or a centralised and planned search-activity, the relevant social groups—that is, the most influential groups—will negotiate the process that ultimately produces a shift within a particular technology or technological assemblage. The authors stress that: “The key point is whether the relevant social groups *see* the problem as being solved” (1987: 44, emphasis in original). Pinch and Bijker’s original propositions certainly met with criticism, arguing that it put too much emphasis on certain actor groups, that the interests guiding these actor groups could not be an *a priori* variable or that it approached an over-simplistic form of social determinism (Hughes 1994; Jasanoff 2004; Klein & Kleinmann 2002). Bijker has since refined the ideas regarding closure and introduced the technological frame concept as a more multi-faceted analytical tool to understand how actors align—or enact controversies—in the shaping of technology. “A technological frame comprises all elements that influence the interactions within relevant social groups and lead to the attributions of meanings to technical artifacts—and thus to constituting technology” (Bijker 1995: 123). The frame thus binds actor groups together around technological constructs through a negotiated process where controversies can be resolved, but does not make up a static or *a priori* relationship.

When controversies appear within a large technical system they can constitute ‘reverse salients’; obstacles that hold back the growth of the system and which, according to Hughes (1992), prompt the system-builder to innovate—but only if the system-builder (a relevant social group) recognises it as a problem. If the relevant social groups do not ‘see’ a problem, no controversy ensues. Then there can be no change in the technological frame.

Recalling the Western experience, water and sanitation technologies had seen many controversies in the nineteenth century. Systems evolved as cities grew, as social fabric was re-woven, ideals and values re-shaped and as economic and scientific capacities expanded. Relevant social groups in Europe and the USA had to negotiate controversies regarding water distribution and pumping technology, piping material, drainage and runoff management, and of course, the development of appliances such as the water closet (Goubert 1989; Reid 1991; Melosi 2000). Wastewater disposal, for instance, caused a long-standing controversy during the nineteenth century. Was it better to discharge sewage water into water bodies or should the use of wastewater for irrigation be encouraged, so as to return valuable fertilizers to the food production chain? Ambitious wastewater irrigation schemes existed in France, Germany and the UK before irrigation gave way for treatment and discharge as land scarcity created closure on this controversy (Reid 1991: 58; Porter 1998: 63). The relevant social groups involved, such as politicians, engineers, planner, doctors, civil rights activists, had partly different objectives and associated different meanings with the technology, which prompted negotiations of the technological frame and led to innovation. Sometimes entirely new and powerful organisations were formed, such as the *London Metropolitan Board of Works* (Halliday 2001).

In many important aspects urban water technologies had already reached closure as they were entering a stage of technology transfer to colonies. Hence, important steps in the negotiation process had not at all taken place in Africa. Colonial system-builders were more inclined to satisfy the preferences of European elites, thus importing and imitating the European solutions, as illustrated above in the case of Kampala. Some adaptation to local conditions took place, but the systems fairly soon stabilised around a piped water and sewerage technology that bore close resemblance to European systems. In Kampala, the technological frame included ideals of modernity along with ample supply of water for subsequent introduction of sewerage, which neither rainwater harvesting nor tapping groundwater sources seemed to offer. The main controversy focused on the financial aspects, where the two most influential social groups, namely the local colonial administrators and the administrators in London held opposing views. Other relevant social groups, such as the Africans, were marginalised in negotiating this process, as they wielded very little power and influence.

Why then, has the Eurocentric “piped paradigm” as Braadbart (2009) has called it, remained so stable even after Independence, though it had proved unable to expand and provide services for the ever-growing urban populations? Certainly not due to the lack of sector reforms. None of these reforms, I would argue, has identified technology itself as the problem. They have typically revolved around economics and institutions, often with a privatization agenda with basis in neoliberal policies (Bayliss 2003; Lobina & Hall 2000; Nilsson & Nyangeri 2008). Water sector reforms have thus been more concerned with emphasising economic viability in the technological frame of large-scale piped systems, rather than stimulating technological change within them. Although reforms have often included a pro-poor agenda, it has seldom been in the interest of international donors and banks to stray too far from the frame within which they know all too well how to operate (Nilsson 2013).

What we really need to understand is why local system builders (governments, city managers and municipal companies) have not actively sought solutions to the obvious misalignment of the systems with local contexts, particularly after decolonisation. Furlong (2014) proposes that the persistent malfunctioning as such has become stabilised and is now considered as something normal. If you do not expect the system to be able to perform any better, then why even try to change it? In combination with a general donor-dependency this may have resulted in a kind of innovation apathy at the level of the local system-builders. The initiative for innovation thus would have come to rest with the donors, supported by the unequal power often associated with the donor-recipient relationship (Ostrom et al. 2002). Two main objections can be made against this hypothesis. First, even if the systems’ underperformance has become stabilised, it still would allow the extension of the same (poor) service level to everyone through redistribution of services and benefits in the system. So far this has not happened, at least not in utilities with persistent malfunctioning like Nairobi, or in Dar es Salaam (Kjellén 2006). Secondly, it fails to explain why the African élites and system builders would have conceded the innovation initiative—and effectively the power over the systems—to new foreign actors so soon after their hard-won independence. All historical evidence points in the opposite direction; that new-born independent governments sought to claim their own authority over these systems.

We may need to look for answers to this conundrum in the long-term fusing of technology and ideology, as well as in power structures in society. As the Mathare Valley case illustrates, a shift of the technological frame towards low-cost solutions threatened to reduce the political value of this frame for central government actors. From the Ministry’s viewpoint, the poor in Mathare were the problem and this could be solved by resettling those “unable to pay” in “agricultural schemes etc.”^45^ The Ministry thus defined the poor as a category of users outside of the urban technological frame. By doing so they reduced a complex reality into schematic categories that supported a particular social and technical order serving the interests of the state, which is precisely what Scott refers to as “state simplification” (1998: 77).

Arguably, the two most influential actor groups in shaping urban infrastructures in the post-independence period in Africa were central government actors—such as ministries of water, housing—and the external financiers, the donors and development banks. To a large extent, these two actor groups had a shared understanding of technology as a vehicle of modernisation. They both ascribed to a common technological frame of urban water supplies as consisting of large-scale capital-intensive piped systems, based on European models, which retained these systems in a perpetual state of closure.

## Conclusion

I started out this paper with a discussion on water technology as a social symbol of progress and of modernisation. I have illustrated how these technical representations of modernity struck a critical chord in the colonisation project. Water and city development become synonymous with colonial expansion as well as a self-legitimising act by virtue of its inherent values of modernism. By creating technological islands of rationality and modernity colonisers dreamt of building sanitary model cities in the tropics. To do so, they used technologies that were essentially ‘closed’ in Pinch and Bijker’s terms and that had been perfected from the Europeans’ point of view. When the colonisers packed their bags in the 1950s and 1960s the new African leaders took up the quest of modernisation and progress, now as a vehicle for African nationalism. And they raised the stakes. Not only did they envisage a new and prosperous Africa; they wished to build their nations using first world technology. “Why should we in modern Ghana be contended with 19^th^ century drains?” as a Ghanaian Member of Parliament put it when foreign experts proposed low-cost and simple technology (Bohman 2010: 99).

In the first years, when economic growth was steadily parked around six to seven per cent per annum, it looked like such an ambitious strategy would win the day. But incentives for cost-recovery eroded in the post-colonial political landscape and within two decades, governments ran out of money (Nilsson & Nyangeri 2008). At this point, the system-builders could have gone back to the drawing board and tried to stimulate technological change and innovation towards technology that was better aligned with socio-economical realities. But adopting lower standards was something that the new leaders, from Ghana to Kenya, obviously saw as a political impossibility. It would have required abandoning ideological positions, which banked on continued progress and modernisation, and a re-moulding of the state machinery inherited from colonialism. Pinch and Bijker (1987) as well as Hughes (1992) have stressed that relevant social groups must first see that there is a problem, before a controversy and subsequent problem-solving can appear. If key decision-makers in Africa, representing the relevant social groups, were more concerned with upholding the image of development and progress, rather than actually delivering service, then it made perfect sense to cling to first-world technologies even when proven ill-suited and incapable of sustainable expansion of service to everyone. Modernity has strong gravity. As James Scott concluded: “Given the ideological advantage of high modernism as a discourse, it is hardly surprising that so many postcolonial elites have marched under its banner” (Scott 1998: 96).

The modernity ideals that once pushed urban infrastructure development in Europe now acted as a conservative force for the very same technologies in African contexts. How is that possible? Tentative explanations could be sought in the governance systems of many African countries. The developmental state was on demise from the 1970s in Africa and was gradually replaced by a minimal and neo-patrimonial state (Walle 2003; Oosterveer 2009). Even if state power—and its finances—was weakening, relevant (and powerful) social groups of the state navigated the landscapes of Africa’s incomplete modernity and found it possible to uphold modern ideals in political rhetoric and in occasional large-scale prestige projects through the state machinery and with the help of donors. But they avoided the obvious controversy of technological change towards cheap and simple solutions as it would have shifted their idea of modernity out of the technological frame. The central governments simply could not see the problem as one that required technological innovation. They saw and they thought along the one dimension that was inscribed in the socio-technical world of modernity surrounding them. They became representatives of what could be called ‘the unseeing state’.

There are strong reasons to believe that innovation, change and adaptation has been severely held back in African cities due to inability of system-builders to see complexities on the ground as well as the need to innovate. It should be noted that the concepts of system-builders and governments are neither monolithic nor homogenous. In the case of Mathare Valley, the initiative to reshape the technological frame of city-building came from local government while actors in central government resisted these attempts. In Nairobi, the view of the central government persevered. The African states may be weak, but a minister still wields more power than a city engineer.

Just like Kooy and Bakker (2008) noted in the case of Indonesia, upholding the image of progress through occasional showcase projects became the model. Bluntly put, African leaders and central government bureaucrats became more interested in political mileage and ribbon-cutting than promoting innovation. The problem they identified was that of securing a steady funding stream from donors for large-scale projects such as the string of large-scale water supply projects for Nairobi (Nilsson 2013). Hence, donors and African governments have helped each other to keep the modern large-scale technology in a state of closure. Political leaders and system-builders are typically not the ones who have been hit by poor water quality or lack of toilets. Weak links of accountability and large inequalities within African societies limit the number of relevant social groups, further reducing pressure for change (Ledant et al. 2013; Drakenberg & Nilsson 2015).

Admittedly, innovative practices like pre-paid water systems and decentralised water systems are now beginning to spread through pilot projects and special initiatives in African cities, often in public-private partnerships (Sharma & Shukla 2009; Heymans et al. 2014). But more wide-spread innovation in large public networks for water and sanitation in the African city will require that relevant social groups—such as water ministry officials, utility managers, experts and donors—see real incentives for change. This could lend strength to an argument for a new privatization agenda, where again market forces will be at play, forcing otherwise ‘unseeing’ or indifferent state actors to innovate. However, with the sobering experience of the 1990s privatization failures and given that water and sanitation were declared human rights in 2010, proposing privatization of water services is not likely to be an option for any reformer.

In the coming decades, African politicians, civic leaders, entrepreneurs and system-builders will need to reshape the ideological framing of water and sanitation technology. Social activism and grass-root movements can demand social justice and more accountable leadership thus creating the necessary pressure for technology change. Such external pressure can be instrumental for shifting the socio-technical landscape and making it more conducive for transformation (Geels 2006). This change is not likely to be abrupt. But as long as water remains technologically a closed topic, other informal, private and small-scale services will continue to grow, capturing ever-increasing market shares from the large-scale public utilities. Perhaps the sustainable future of the African city is harboured in alternative, small-scale and people-centred technology. Some of these small-scale and multi-centric solutions—sometimes known as “inverse infrastructures”—are already challenging large-scale and centralist models in other parts of the world in areas like energy, solid waste and information and communications technology (Egyedi & Mehos 2012).

We are just beginning to understand socio-technical change in African cities. Detailed and wide-ranging historical analysis of cities, people and their infrastructure in Africa will allow a more nuanced and clearer picture of where we are, and how transformation towards sustainability can take place. We need to know how the negotiation process between the relevant social groups has played out in detail, to better assess what changes in the social landscape can create positive incentives for technology change and innovation. Perhaps such insights could facilitate a shift in governments’ policies and practices, turning the ‘unseeing state’ into one that allows and encourages key actors to start seeing problems on the ground in a new and more change-oriented way. Comparative analyses between countries and between infrastructure areas should also be vital. Most of all, we need to challenge our own thinking about what is right and what is possible in the context of Africa’s urban revolution. To do this we must, as Fourchard’s (2011) suggests, dismantle our implicit comparisons with the North. In the early twenty-first century, the African city is the place to be for anyone interested in technology change and social transformation. The rest of the world is likely to learn a lot from Africa in the coming decades.

## Footnotes

1 Data for 1963 is from World Health Organisation (WHO) 1963. “The organization for water development in Kenya, Report of a World Health Organisation Consultant Team”, Kenya National Archives (KNA) reference: BY/138/43. Data for 2009 is from Kenya National Bureau of Statistics, 2009 Population and Housing Census report. The claim for universal coverage in 1963 was based on official statistics provided by the outgoing colonial administration and may be somewhat overstated. However, the official figure can be expected to be high as urban migration had been strictly regulated during colonial times, making most of the settlement taking place in planned and thus serviced areas.

2 The discussion on privatization of water is extensive, see for instance Rogers et al. (1998); Lobina & Hall (2000); Gleick et al. (2002); Budds & McGranahan (2003); Dellapenna (2009).

3 The resolution of water and sanitation as human rights: United Nations, 2010. General Assembly Resolution A/64/L.63/Rev.1, 26/7/2010.

4 Schumacher (1973); Edquist & Edkvist (1978); Therkildsen (1988); Vaa (1993); Black (1998); Tilley et al. (2008).

5 See e.g. Winner (1980); Noble (1984); Hecht (1998).

6 Uganda Protectorate, “Medical and Sanitary Report for 1926”, UK National Archives (UKNA) CO/685/10.

7 Colonial Office 1915, “Report on sanitary matters in the East Africa protectorate, Uganda, and Zanzibar, East Africa”, KNA Library, GP 363.7 SIM.

8 Uganda Protectorate, not dated [1924]. “Proposed water supply for Kampala and Jinja—Interim Report by the Executive Engineer Water Supply.” Enclosed in Jarvis to Colonial Office 4 September 1926, UKNA, CO/536/143/6.

9 Acting Governor Jarvis to Colonial Office UK, dated 4 September 1926, UKNA, CO/536/143/6. Includes a report from Engineer Morris on Kampala Water Supply.

10 Secretary of State Mr. Amery to Uganda Protectorate, with enclosure, dated 28 October 1926, UKNA, CO/536/143/6.

11 Acting Governor Mr. Jarvis to Colonial Office, dated 26 January 1927, UKNA, CO/536/145/3.

12 Governor Gowers to Colonial Office, dated 7 October 1927, UKNA, CO/536/145/3.

13 Uganda Protectorate, “Report on Kampala Water Supply”, dated March 1927, enclosed in Gov. Gowers to Colonial Office, 7 October 1927.

14 Secretary of State Mr. Avery to Governor Gowers, draft dated 20 August 1928, UKNA, CO/536/148/6.

15 Uganda Protectorate, “Annual report of the Medical Department for 1936”, UKNA, CO/685/21.

16 Director of Public Works to Chief Secretary Uganda, dated 11 July 1927, enclosed in Gov. Gowers to Colonial Office, 7 October 1927.

17 Governor Gowers dispatch to the Colonial Office, UK, dated 7 January 1929, UKNA, CO/536/153/3.

18 East Africa Protectorate 1913. “Nairobi Sanitary Commission 1913”. KNA, K 363.72, p. 13.

19 British East Africa Protectorate 1907. “Report on the Sanitation of Nairobi”, dated 28 January 1907, KNA GP 363.7. BRI; East Africa Protectorate 1913, Nairobi Sanitary; Colonial Office 1915, “Report on sanitary matters.”

20 British East Africa Protectorate 1917, Report, p. 18.

21 Smart J. 1950. “A Jubilee History of Kenya”, KNA, GP 307.76 SMA.

22 East African Standard, 17 November 1938, “Nairobi’s Water. Governor opens filtration plant”, KNA BY/19/295.

23 Colony and Protectorate of Kenya 1934. “Report. Ruiru water supply scheme”. KNA, RN/1/88.

24 Colony and Protectorate of Kenya 1945. “Report on augmentation of water supply for the Nairobi municipal area”, KNA BY/19/295.

25 Colony and Protectorate of Kenya 1948. “Annual Report for 1946”, Nordic Africa Institute Library, Uppsala.

26 Colony of Kenya, “Report of the Commissioner for local government for the year 1956”, KNA Library K 352. 06762 0005 KEN.

27 Colony and Protectorate of Kenya 1934. “Ruiru water supply”. The term “substituted natives” refers to those groups in the African population that were deemed to have sufficient education to gradually replace—substitute—Indians within the general workforce.

28 Republic of Kenya 1965. “African Socialism and its Application to Planning in Kenya. Sessional Paper no.10.” Government printer: Nairobi, p. 1. Nordic Africa Institute Library.

29 Republic of Kenya 1965 p. 8.

30 Republic of Kenya 1970. “Development Plan 1970–1974”. Government Printer: Nairobi. Nordic Africa Institute Library.

31 Nairobi City Council (NCC). “Mathari valley. Squatters meeting. Minutes of meeting held 7 January 1969”, KNA RN/6/21.

32 Letter from J. Kamau, National Christian Church of Kenya to Town Clerk, Nairobi City Council, dated 13 February 1970, KNA RN/6/21.

33 NCC, City Engineer. “Mathare valley project. Report to the special meeting of social services and housing committee^”^, June 1969, KNA RN/6/21.

34 Draft letter, permanent secretary of Min. of Housing to permanent secretary of Treasury, Mathari Valley resettlement scheme, not dated [1969], KNA ref. KY/3/6.

35 Republic of Kenya, Ministry of Housing. Minister to permanent secretary, “Top priority”, 7 June 1969; permanent secretary Min. of Housing to permanent secretary Treasury, “Urban renewal in Nairobi”, 9 June 1969, KNA ref. KY/3/6.

36 Daily Nation, “Homes pledge to Mathare”, 6 June 1969, KNA KY/3/6.

37 Republic of Kenya, Ministry of Housing, “Notes of a meeting on a settlement of squatters from Mathare valley held in Jogoo House 29 May 1969”, KNA KY/3/6.

38 NCC, City Engineer, “Mathare valley project. Report to the special meeting of social services and housing committee”, June 1969, KNA RN/6/21.

39 NCC, City Engineer, “Mathare valley project. Report to the special meeting of social services and housing committee”, June 1969, KNA RN/6/21.

40 L. Laurentii, adviser to Ministry of Economic Planning and Development, to permanent secretary of Ministry of Housing Mr. J N Oluoch, “Mathare Valley resettlement”, 17 June 1969. KNA KY/3/6.

41 NCC, City Engineer, “Mathare valley project. Report to the special meeting of social services and housing committee”, June 1969, KNA RN/6/21.

42 NCC, “Mathare valley housing development project report 1974”, October 1974, KNA RN/6/70.

43 EGIS BCEOM, “Feasibility study and master plan for developing new sources for Nairobi and satellite towns”, report to Athi Water Service Board, 6 September 2011.

44 Bo Sandgren, personal communication 2007. Sandgren was chief technical adviser to Kampala City Council during implementation of the project. The author was at the time programme officer at Sida, in charge of the financing of the pilot project.

45 “Notes of a meeting on a settlement of squatters from Mathare valley held in Jogoo House 29 May 1969”, KNA KY/3/6.
